# Hit and Indexing Rate in Serial Crystallography: Incomparable Statistics

**DOI:** 10.3389/fmolb.2022.858815

**Published:** 2022-03-25

**Authors:** Ki Hyun Nam

**Affiliations:** ^1^ Department of Life Science, Pohang University of Science and Technology, Pohang, South Korea; ^2^ POSTECH Biotech Center, Pohang University of Science and Technology, Pohang, South Korea

**Keywords:** serial crystallography, data processing, hit rate, indexing rate, data statistics

## Introduction

Serial crystallography (SX) using X-ray free-electron lasers (XFEL) and synchrotron X-rays is an emerging X-ray crystallography technique to determine the structure of macromolecules at room temperature or near-physiological temperature with minimal radiation damage (Chapman et al., 2011; Boutet et al., 2012; Chapman et al., 2014; Stellato et al., 2014; Johansson et al., 2017; Standfuss and Spence, 2017; Nam, 2019; Nam, 2021b; Durdagi et al., 2021; Nam, 2022c). This technique is used for studying time-resolved molecular mechanisms through pump-and-probe experiments with an optical laser or a liquid application (e.g., substrate or inhibitors) (Spence, 2014; Schulz et al., 2018; Schmidt, 2019; Butryn et al., 2021; Martin-Garcia, 2021). The SX technique overcomes the experimental limitations of traditional X-ray crystallography. This technique causes minimal radiation damage, does not need a cryogenic environment, and provides dynamic structural information; furthermore, it provides biologically relevant structural information with accurate visuals depicting the molecular mechanism (Chapman et al., 2011; Boutet et al., 2012; Chapman et al., 2014; Schmidt, 2019; Orville, 2020; Pearson and Mehrabi, 2020; Nam, 2021a; Nam, 2022c).

**FIGURE 1:**
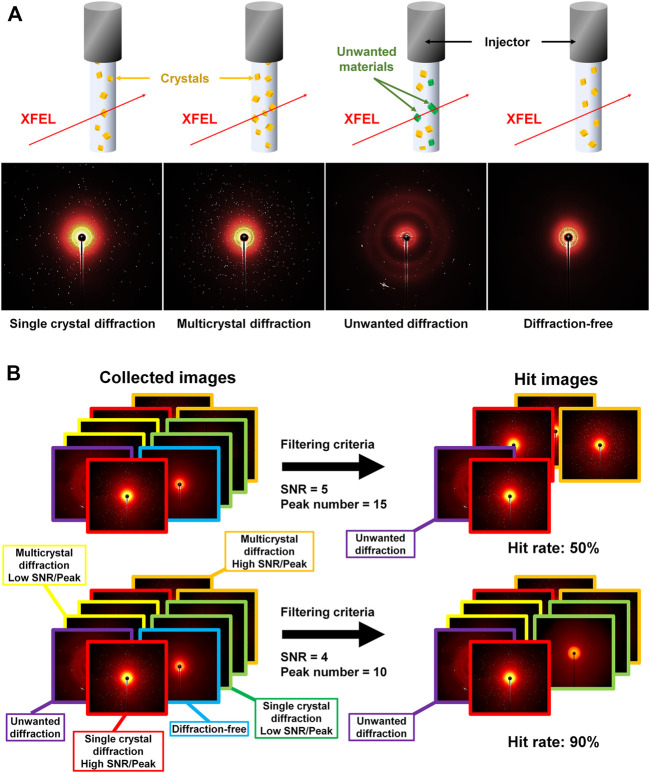
**(A)** Examples of collected image in serial crystallography: single crystal diffraction, multicrystal diffraction, unwanted material diffraction, and diffraction-free images. **(B)** Example of change in hit rate according to the hit filtering parameter. Single crystal diffraction (high SNR/Bragg peak number), single crystal diffraction (low SNR/Bragg peak number), multiple crystal diffraction (high SNR/Bragg peak number), multiple crystal diffraction (low SNR/Bragg peak number), unwanted diffraction (for example, salt), and non-diffraction images are indicated by images outlined in red, yellow, orange, green, purple, and blue, respectively.

In an SX experiment, a large number of crystals are serially delivered to an X-ray interaction point via various sample delivery techniques, such as injectors injector (DePonte et al., 2008; Weierstall et al., 2014), syringes with viscous medium (Sugahara et al., 2015; Park and Nam, 2019; Nam, 2020a; Nam, 2022a), fixed-target scanning (Hunter et al., 2014; Murray et al., 2015; Lee et al., 2019; Lee et al., 2020; Park et al., 2020; Nam et al., 2021), capillaries (Stellato et al., 2014; Nam, 2020b), convey belts (Beyerlein et al., 2017a), and microfluidics (Knoska et al., 2020; Monteiro et al., 2020; Nam and Cho, 2021). Crystals are exposed to X-rays only once for a short period of time at the XFEL (fs level) or synchrotron (ms level). A large number of images (ranging from thousands to millions) are collected to determine the three-dimensional structure of macromolecules during SX data collection (Schmidt, 2019). Delivering the crystals spatiotemporally in a continuous manner at the X-ray interaction location during SX data collection is experimentally impossible. Hence, the collected data include images that contain diffraction information generated while penetrating X-ray crystals and other images that do not penetrate the crystal. In general, four types of images can be collected, as follows: 1) single crystal diffraction, 2) multicrystal diffraction, 3) unwanted diffraction or scattering (salt or crystal delivery materials), and 4) diffraction-free images (Figure 1A).

In SX technology, a “hit” denotes a diffraction pattern with the minimum number of detectable Bragg peaks (Barty et al., 2014). As only hit images containing Bragg peaks are needed for structure determination, hit images are filtered from whole images using image filtering programs and employed for the next data processing step. Filtering the hit image has the following two advantages: 1) Filtering only hit images reduces the time needed for the next data processing step and aids in the efficient utilization of available computing resources. 2) Excluding the non-hit images reduces storage consumption and file conversion time (e.g., cxi to hdf5). Meanwhile, the hit rate (ratio) is obtained by dividing the number of hit images by the total number of images collected. This hit rate provides primary information about the number of images suitable for data processing and the diffraction quality and density of crystals during SX data collection. This information can be used for preparing samples and determining the data collection efficiency.

Bragg peaks are indexed from the hit images including the diffraction pattern to obtain information regarding three integers (*h*, *k*, and *l*) (Otwinowski and Minor, 1997). Subsequently, Bragg peaks are integrated and scaled to obtain the structure factor. Indexed images refer to images in which the input unit cell parameter and information about the crystal system match. The indexing rate (ratio) is a statistic obtained by dividing the number of indexed images that match the input crystal information by the total number of hit images. Therefore, the indexing rate can provide information about the crystal and data quality during data collection and processing.

The hit rate and indexing rate provide information about the crystal density and crystal quality, respectively, used during data collection and aid in calculating the amount of data sufficient for determining the crystal structure or changing the experimental parameter. This information aids in utilizing the beamtime efficiently. Meanwhile, SX researchers and journal reviewers/editors often evaluate and compare the hit rate and indexing rate numbers of independent SX experiments. However, the hit rate and indexing rate of independent SX experiments cannot be compared because the rates can represent distinct values depending on the experimental results or program parameters. Moreover, the hit rate and indexing rate can be increased or decreased easily by altering the settings of the data processing program. Accordingly, I believe the hit rate and indexing rate are just statistics that cannot be compared with independent experiments.

## Discussion

### Hit Rate

The hit rate is an important statistic for determining the data acquisition efficiency and planning beamtime utilization in experiments. For example, when the crystal hit rate is low during data collection, researchers can replace the sample with fresh crystals or increase the crystal density, which may increase the hit rate and yield more hit images containing the diffraction pattern for the remaining beamtime. Meanwhile, although obtaining a large number of hit images is important to increase the SX data collection efficiency, when the crystal hit rate is high with intense multiple crystal diffraction patterns during data collection, researchers may decrease the density of the crystal sample. This reduces the hit rate, but it offers the advantage of avoiding the incorrect indexing of the Bragg peaks and the incorrect signal-to-noise ratio (SNR) related to the background noise.

Crystal density is calculated based on the sample delivery method (e.g., sample volume) and X-ray properties (e.g., exposure time, repetition rate, and beam size) to obtain an appropriate hit rate. The crystals are delivered continuously to an X-ray location to collect diffraction data. In an ideal experiment, new crystals (or larger crystals with a new volume) would be delivered continuously at every X-ray exposure point, resulting in a 100% hit rate. However, providing crystals precisely each time both spatially and temporally through which X-rays are transmitted is experimentally impossible. The collected SX data include the diffraction image in which X-rays pass through the crystal and the image information in which the crystal is not hit. In addition, unwanted diffraction from salt crystals and the sample delivery material may occur experimentally during data acquisition. This unwanted diffraction can be sorted as a Bragg peak and processed as a hit image by the filtering program, leading to an increase in the hit rate.

Programs such as Cheetah (Barty et al., 2014), NanoPeakCell (Coquelle et al., 2015), and Psocake (Thayer et al., 2017) can be used to filter hit images from the collected SX data. These programs filter hit images that meet the criteria for selection as hit images, including parameters such as the number of Braggs peaks, minimum SNR, and number of connected pixels above the minimum SNR. These filtering parameters can affect the number of hit images, as researchers can change settings based on data quality (Figure 1B). For example, if researchers lower the criteria for filtering parameters such as the SNR and peak number to include low Bragg peak intensities, the hit rate will increase. Conversely, if the researchers raise the criteria for the filtering parameters to only use data with high Bragg peak intensities, the hit rate will be lower. Therefore, hit rates are variables that can exhibit differences based not only on sample quality but also on the filtering program settings. Hence, a direct correlation between data collection efficiency and hit rate cannot be established. Therefore, hit rates of independent experiments cannot be compared and evaluated.

### Indexing Rate

The hit images including the Bragg peaks are indexed, integrated, and scaled to provide the final three-dimensional structural information. The accurate indexing of crystal diffraction patterns in the first data processing step is essential to provide an accurate structure factor. In general, higher indexing rates provide better data statistics in terms of using more diffraction patterns. Factors affecting the indexing rate include the quality of the acquired image, optimization of the detector geometry, indexing program used, and technical skills. In terms of data quality, the following factors can decrease the indexing rate: 1) several space groups of crystal forms existing in the crystal sample, 2) Bragg peaks with low SNR levels, 3) salt peaks or unwanted intensities, and 4) mis-indexing because of multicrystal diffraction patterns.

Information about the detector geometry, including the X-ray energy, crystal-to-detector distance, and detector specifications is essentially required to index the diffraction patterns from hit images in the SX experiment. The indexing efficiency varies based on the accuracy of the detector geometry information. For example, segmented detectors consist of several small detector modules tiled together, such as Cornell-SLAC Pixel Array Detectors (CSPAD) (Moeller et al., 2012), multi-port charge-coupled devices (MPCCD) (Kameshima et al., 2014), adaptive gain integrating pixel detectors (AGIPD) (Allahgholi et al., 2019), Percival (Marras et al., 2019), and adJUstiNg Gain detector FoR the Aramis User station (JUNGFRAU) (Leonarski et al., 2020) detectors. Geometry optimization may be necessary for each panel during data processing because the pixels in each module may not be perfectly aligned on a regular grid. A previous geometry study showed that the indexing rate of Gd:lysozyme, cathepsin B, DgkA, and rhodopsin-arrestin data sets collected from different SX experiments were improved by 3–60% after geometry refinement (Yefanov et al., 2015). Therefore, geometric optimization is required for efficient indexing of diffraction patterns, and the indexing rate may differ depending on the accuracy of the detector geometry optimization.

Moreover, the indexing rate may vary depending on the indexing programs used for data processing, indexing algorithms, or indexing parameters (Nam, 2022b). Currently, various indexing programs such as CrystFEL (White et al., 2016; White, 2019), *dials. index* in DIALS (Gildea et al., 2014), Computational Crystallography Toolbox (cctbx) (Brewster et al., 2015), FELIX (Beyerlein et al., 2017b), SPIND (Li et al., 2019), XGANDALF (Gevorkov et al., 2019), Pattern-matching indexing (Dejoie and Tamura, 2020), SPIND-TC (Li et al., 2020) and MCDPS (Zhou et al., 2021) have been developed for SX data analysis, and they analyze diffraction patterns using their unique approaches with various algorithms. Each of these indexing algorisms exhibits different indexing rates and data statistics even when processed using the same indexing parameters, including the detector geometry. Furthermore, the indexing rate can be increased using a combination of several indexing algorithms, which may provide good statistical values with a high indexing rate. However, this does not necessarily result in better structure refinement statistics. In addition, the indexing rate changes during data processing optimization according to the changes in the indexing parameters (e.g., unit cell parameter tolerance, SNR cutoff, and integration radius).

Consequently, the indexing rate varies depending on the quality of the collected data, program used, - technical skills of the individual during processing, and setting of the indexing parameters, even when the procedure for indexing the Bragg peaks in a diffraction pattern is the same. Meanwhile, in general SX data processing, researchers process data by increasing the indexing rate; however, if sufficient diffraction images are collected, increasing the indexing standard and using only excellent data will provide better structural information. On the other hand, since the structure factor is obtained from the correctly indexed images, more important feedbacks than the hit rate during experiments are the accumulated numbers or increasing rate of valid images (indexable patterns).

## Conclusion

In the SX experiment, the hit rate and indexing rate can be used to evaluate the sample quality, data collection strategy, and beamtime efficiency during data collection and processing. However, these rates can be increased or decreased according to the processing parameters used. Hence, hit rate and indexing rate cannot be used to analyze the SX experimental results.
